# Molecular characterization of clinical carbapenem-resistant *Enterobacterales* from Qatar

**DOI:** 10.1007/s10096-021-04185-7

**Published:** 2021-02-22

**Authors:** Fatma Ben Abid, Clement K. M. Tsui, Yohei Doi, Anand Deshmukh, Christi L. McElheny, William C. Bachman, Erin L. Fowler, Ahmed Albishawi, Kamran Mushtaq, Emad B. Ibrahim, Sanjay H. Doiphode, Manal M. Hamed, Muna A. Almaslmani, Abdullatif Alkhal, Adeel A. Butt, Ali S. Omrani

**Affiliations:** 1 Division of Infectious Diseases, Department of Medicine, Hamad Medical Corporation, Doha, Qatar; 2 Communicable Diseases Center, Hamad Medical Corporation, PO Box 3050, Doha, Qatar; 3 Weill Cornell Medicine-Qatar, Doha, Qatar; 4 Department of Pathology, Sidra Medicine, Doha, Qatar; 5 Division of Infectious Diseases, Faculty of Medicine, University of British Columbia, Vancouver, Canada; 6 Division of Infectious Diseases, University of Pittsburgh School of Medicine, Pittsburgh, PA USA; 7 Departments of Microbiology and Infectious Diseases, Fujita Health University School of Medicine, Toyoake, Japan; 8 Division of Microbiology, Department of Pathology and Laboratory Medicine, Hamad Medical Corporation, Doha, Qatar; 9 Department of Medicine, Hamad Medical Corporation, Doha, Qatar; 10 Clinical Epidemiology Research Unit, Hamad Medical Corporation, Doha, Qatar

**Keywords:** CRE, Carbapenemase, KPC, NDM, VIM, OXA, Enterobacterales, Qatar, Middle East

## Abstract

One hundred forty-nine carbapenem-resistant *Enterobacterales* from clinical samples obtained between April 2014 and November 2017 were subjected to whole genome sequencing and multi-locus sequence typing. *Klebsiella pneumoniae* (81, 54.4%) and *Escherichia coli* (38, 25.5%) were the most common species. Genes encoding metallo-β-lactamases were detected in 68 (45.8%) isolates, and OXA-48-like enzymes in 60 (40.3%). *bla*_NDM-1_ (45; 30.2%) and *bla*_OXA-48_ (29; 19.5%) were the most frequent. KPC-encoding genes were identified in 5 (3.6%) isolates. Most common sequence types were *E. coli* ST410 (8; 21.1%) and ST38 (7; 18.4%), and *K. pneumoniae* ST147 (13; 16%) and ST231 (7; 8.6%).

**Supplementary Information:**

The online version contains supplementary material available at 10.1007/s10096-021-04185-7.

## Introduction

Considerable variations exist in the epidemiology of carbapenemases in *Enterobacterales* from different parts of the world [1]. Awareness of the locally prevalent carbapenemases is relevant to the appropriate selection of antimicrobial therapy for carbapenem-resistant *Enterobacterales* (CRE) infections [2]. Moreover, the molecular epidemiology of CRE could help guide control efforts. The aim of this study was to identify the predominant carbapenemases in CRE from Qatar and to elucidate their molecular epidemiology.

## Methods

All carbapenem-resistant *Enterobacterales* isolates from clinical specimens received at Hamad Medical Corporation Microbiology Department during the period between April 2014 and November 2017 were included. The department provides diagnostic microbiology services for all public hospitals in Qatar. Laboratory methods for bacterial identification, antimicrobial susceptibility testing, whole genome sequencing and analysis are described in the supplementary data file.

## Results

A total of 149 non-repetitive CRE clinical isolates from 146 patients were included. The median patient age was 57 years (range, 3 months–97 years), and 77 (52.7%) were males. Diabetes mellitus (73; 50%) and chronic kidney diseases (39; 26.7%) were the most frequent co-existing medical conditions. Nineteen (12.8%) isolates were recovered from samples obtained within the first 24 h of hospitalization. Nearly one-fifth of the patients (25, 17.1%) had entered the country from overseas within 30 days prior to CRE isolation. Further demographic and clinical details are provided in Table 1 and in Table S1 in the supplementary data file.

**Table 1:** Patient demographics and clinical outcome

Variable	Number (%) (149 isolates from 146 patients)
Demographics	
Median age in (range)	57 years (3 months–97 years)
Male sex	77 (52.7%)
Nationality of origin*	
Eastern Mediterranean Region	96 (65.7%)
South East-Asia Region	41 (28.1%)
European Region	3 (2%)
Regions of the Americas	1 (0.7%)
Comorbidities	
Diabetes mellitus	73 (50%)
Chronic kidney disease	39 (26.7%)
Congestive heart failure	20 (13.7%)
Chronic liver disease	9 (6.1%)
Chronic airway disease	7 (4.8%)
Solid malignancy	20 (13.7%)
Hematological malignancy	11 (7.5%)
Solid organ transplant	11 (7.5%)
Hematopoietic stem cell transplant	4 (2.7%)
Active chemotherapy within 30 days of CRE isolation	16 (10.9%)
Anti-TNF therapy within 30 days of CRE isolation	15 (10.3%)
Corticosteroid therapy within 30 days of CRE isolation	15 (10.3%)
Carbapenem therapy within 30 days of CRE isolation	45 (30.8%)
Invasive procedure within 30 days of CRE isolation	
Surgical procedure	41 (28.1%)
Central line insertion	29 (19.8%)
Urinary catheterization	29 (19.8%)
Vascular stent insertion	11 (7.5%)
Other risk factors for CRE infection	
Presence of central venous catheter	42 (28.7%)
Total parenteral nutrition	5 (3.4%)
Mechanical ventilation	41 (28.1%)
Travel within 30 days of CRE isolation	25 (17.1%)
Site of isolation	
Urinary tract	41 (28.1%)
Blood stream	33 (22.6%)
Lower respiratory tract	17 (11.6%)
Skin and soft tissue	8 (5.5%)
Others	14 (9.6%)
Clinical outcome	
Death within 30 days from CRE isolation	34 (23.3%)
Recurrence with 30 days	38 (26%)

*World Health Organization Regions. *CRE*, carbapenem-resistant *Enterobacterales*

The most frequent species were *Klebsiella pneumoniae* (81; 54.4%) and *Escherichia coli* (38; 25.5%) (Table 2), whereas urine (41; 27.5%) and blood (33; 22.1%) were the most frequent site of isolation (Table 1). Thirty-four (23.3%) patients died within 30 days of first CRE isolation. The majority of isolates were susceptible to tigecycline (129; 86.5%), fosfomycin (126; 84.6%), and amikacin (108; 72.4%) (Table S2 in the supplementary data file).

**Table 2:** Carbapenemase genes detected in the study isolates

Carbapenemase genes	All isolates (*n* = 149)	*E. coli* (*n* = 38)	*K. pneumoniae* (*n* = 81)	*K. quasipneumoniae* (*n* = 16)	*E. cloacae* (*n* = 7)	Others* (*n* = 7)
Class A	4 (2.7%)	0	4 (4.9%)	0	0	0
*bla*_KPC-2_	3 (2.0%)	-	3 (3.7%)	-	-	-
*bla*_KPC-3_	1 (0.7%)	-	1 (1.2%)	-	-	-
Class B	64 (43.0%)	14 (36.8%)	24 (29.6%)	16 (100%)	6 (85.7%)	4 (57.1%)
*bla*_IMP-26_	1 (0.6%)	1 (2.6%)	-	-	-	-
*bla*_NDM-1_	45 (30.2%)	3 (7.9%)	22 (27.2%)	14 (87.5%)	4 (57.1%)	2^†^ (28.6%)
*bla*_NDM-5_	7 (4.7%)	7 (18.4%)	-	-	-	-
*bla*_NDM-6_	1 (0.7%)	1 (2.6%)	-	-	-	-
*bla*_NDM-7_	5 (3.4%)	-	1 (1.2%)	2 (12.5%)	-	2^‡^ (28.6%)
*bla*_NDM-13_	1 (0.7%)	1 (2.6%)	-	-	-	-
*bla*_NDM-19_	1 (0.7%)	1 (2.6%)	-	-	-	-
*bla*_VIM-2_	1 (0.7%)	-	1 (1.2%)	-	-	-
*bla*_VIM-4_	2 (1.3%)	-	-	-	2 (28.6%)	-
Class D	59 (39.6%)	21 (55.2%)	34 (42%)	0	1 (14.3%)	2 (28.6%)
*bla*_OXA-48_	29 (19.5%)	10 (26.3%)	16 (19.8%)	-	1 (12.3%)	2^§^ (28.6%)
*bla*_OXA-181_	19 (12.8%)	10 (26.3%)	9 (11.1%)	-	-	-
*bla*_OXA-232_	10 (6.7%)	-	10 (12.3%)	-	-	-
*bla*_OXA-244_	1 (0.7%)	1 (2.6%)	-	-	-	-
Multiple carbapenemases	8 (5.3%)	1 (2.6%)	7 (8.6%)	0	0	1 (14.3%)
*bla*_NDM-1_ + *bla*_KPC-3_	1 (0.7%)	-	1 (1.2%)	-	-	-
*bla*_NDM-1_ + *bla*_OXA-181_	2 (1.3%)	-	2 (2.5%)	-	-	-
*bla*_NDM-5_ + *bla*_OXA-48_	3 (2.0%)	-	3 (3.7%)	-	-	-
*bla*_NDM-5_ + *bla*_OXA-181_	2 (1.3%)	1 (2.6%)	1 (1.2%)	-	-	-
None	14 (9.4%)	1 (2.6%)	12 (14.8%)	-	-	1¶ (12.3%)

*Klebsiella aerogenes (2), *Providencia* spp. (2), *Citrobacter freundii* (1), *Klebsiella oxytoca* (1), and *Proteus mirabilis* (1)

^†^*Providencia* sp. (1), *P. mirabilis* (1)

^‡^*C. freundii* (1), *Providencia* sp. (1)

^§^*K. oxytoca* (1), *K. aerogenes* (1)

^¶^*K. aerogenes* (1)

Genes encoding Class B carbapenemases (i.e., metallo-β-lactamases [MBL]) were detected in 68 (45.8%) isolates, and Class D carbapenemases in 60 (40.3%). The most frequently identified carbapenemase genes were *bla*_NDM-1_ (45; 30.2%) and *bla*_OXA-48_ (29; 19.5%) (Table 3). KPC-encoding genes were identified in 5 (3.6%) isolates. All 5 patients from whom KPC-producing *K. pneumoniae* were isolated had received medical care abroad within the preceding 30 days (Table S3 in the supplementary data file). No carbapenemase genes were detected in 14 (9.4%) isolates, and 8 (5.3%) carried more than one carbapenemase genes (Table 2).

**Table 3:** Study isolates by their sequence type and carbapenemase genes

Species	Sequence type (number of isolates)	Carbapenemase-encoding genes (number of isolates)
*E. coli* (*n* = 38)	ST10 (2)	*bla*_NDM-5_ (1), none (1)
	ST23 (1)	*bla*_OXA-48_ (1)
	ST38 (7)	*bla*_OXA-48_ (6), *bla*_OXA-181_ (1)
	ST58 (1)	*bla*_OXA-48_ (1)
	ST101 (2)	*bla*_NDM-1_ (1), none (1)
	ST131 (1)	*bla*_IMP-26_ (1)
	ST167 (3)	*bla*_NDM-1_ (1), *bla*_NDM-13_ (1), *bla*_NDM-19_ (1)
	ST361 (1)	*bla*_NDM-5_ (1)
	ST405 (2)	*bla*_OXA-48_ (1), *bla*_NDM-5_ (1)
	ST410 (8)	*bla*_OXA-181_ (7)_,_ *bla*_NDM-5_ and *bla*_OXA-181_ (1)
	ST617 (2)	*bla*_NDM-5_ (2)
	ST648 (2)	*bla*_OXA-48_ (1), *bla*_OXA-244_ (1)
	ST940 (1)	*bla*_NDM-1_ (1)
	ST1487 (2)	*bla*_NDM-5_ (1), *bla*_OXA-181_ (1)
	ST1193 (1)	*bla*_NDM-5_ (1)
	ST8346 (1)	*bla*_OXA-181_ (1)
	Undetermined (1)	*bla*_NDM-6_ (1)
*K. pneumoniae* (*n* = 81)	ST4 (1)	*bla*_NDM-1_ (1)
	ST10 (2)	*bla*_NDM-5_ (1), none (1)
	ST11 (5)	*bla*_NDM-1_ (4), *bla*_KPC-2_ (1)
	ST14 (2)	*bla*_NDM-1_ (2)
	ST15 (2)	*bla*_NDM-1_ (1), *bla*_KPC-2_ (1)
	ST16 (3)	*bla*_NDM-1_ + *bla*_OXA-181_ (1), *bla*_OXA-181_ (1), *bla*_OXA-232_ (1)
	ST17 (1)	*bla*_NDM-1_ (1)
	ST35 (1)	*bla*_OXA-181_ (1)
	ST38 (1)	*bla*_OXA-232_ (1)
	ST39 (3)	*bla*_NDM-1_ (3)
	ST101 (1)	None (1)
	ST147 (13)	*bla*_NDM-1_ (4), *bla*_OXA-181_ (5), *bla*_OXA-48_ (1), *bla*_NDM-1_ + *bla*_OXA-181_ (1), *bla*_NDM-5_ + *bla*_OXA-181_ (1), none (1)
	ST220 (1)	None
	ST231 (7)	*bla*_OXA-232_ (4), *bla*_OXA-48_ (1), none (2)
	ST252 (1)	*bla*_NDM-1_ (1)
	ST258 (1)	*bla*_KPC-3_ (1)
	ST280 (1)	*bla*_OXA-48_
	ST307 (2)	None (2)
	ST323 (1)	*bla*_OXA-48_
	ST340 (2)	*bla*_NDM-1_ (2)
	ST376 (1)	*bla*_OXA-48_
	ST383 (4)	*bla*_OXA-48_ (1), *bla*_NDM-5_ + *bla*_OXA-48_ (3)
	ST395 (2)	*bla*_OXA-232_ (1), none (1)
	ST405 (1)	*bla*_OXA-48_ (1)
	ST420 (2)	*bla*_OXA-48_ (2)
	ST432 (1)	*bla*_OXA-48_ (1)
	ST584 (1)	None (1)
	ST716 (2)	*bla*_OXA-48_ (2)
	ST873 (1)	*bla*_OXA-48_ (1)
	ST915 (3)	*bla*_NDM-1_ (3)
	ST983 (1)	*bla*_OXA-48_ (1)
	ST985 (1)	None (1)
	ST1193 (2)	*bla*_OXA-48_ (1), *bla*_NDM-7_ (1)
	ST1418 (1)	*bla*_OXA-181_ (1)
	ST1626 (1)	*bla*_OXA-48_ (1)
	ST2096 (2)	*bla*_OXA-232_ (2)
	ST3311 (1)	None (1)
	ST5029 (1)	*bla*_KPC-2_ (1)
	ST5030 (1)	*bla*_OXA-232_ (1)
	ST5031 (1)	*bla*_OXA-181_ (1)
	Undetermined (2)	*bla*_KPC-3_ + *bla*_NDM-1_ (1), *bla*_VIM-2_ (1)
*K. quasipneumoniae* (*n* = 16)	ST196 (9)	*bla*_NDM-1_ (9)
	ST1416 (4)	*bla*_NDM-1_ (4)
	ST1584 (1)	*bla*_NDM-7_ (1)
	ST1998 (1)	*bla*_NDM-7_ (1)
	Undetermined (1)	*bla*_NDM-1_ (1)
*E. cloacae* (*n* = 7)	ST66 (1)	*bla*_OXA-48_ (1)
	ST113 (1)	*bla*_NDM-1_ (1)
	ST511 (1)	*bla*_NDM-1_ (1)
	ST609 (1)	*bla*_NDM-1_ (1)
	ST78 (3)	*bla*_NDM-1_ (1), *bla*_VIM-4_ (2)
*K. aerogenes* (*n* = 2)	ST93 (1)	None (1)
	Undetermined (1)	*bla*_OXA-48_ (1)
*Providencia* sp. (*n* = 2)	Undetermined (1)	*bla*_NDM-1_ (1), *bla*_NDM-7_ (1)
*C. freundii* (*n* = 1)	Undetermined (1)	*bla*_NDM-7_ (1)
*K. oxytoca* (*n* = 1)	Undetermined (1)	*bla*_OXA-48_ (1)
*P. mirabilis* (*n* = 1)	Undetermined (1)	*bla*_NDM-1_ (1)

The included *E. coli* isolates belonged to 16 different sequence types, the most frequent of which were ST410 (8; 21.1%) and ST38 (7; 18.4%). On the other hand, *K. pneumoniae* isolates represented 40 different sequence types, most frequently ST147 (13; 16%) and ST231 (7; 8.6%). *K. quasipneumoniae* belonged mainly to ST196 (9; 56.3%) and ST1416 (4; 25%). Except for *K. quasipneumoniae*, strains with the same sequence type mostly possessed different carbapenemase genes (Table 3).

## Discussion

Typical risk factors for multidrug-resistant Gram-negative infections were highly prevalent in the patients from whom the isolates were retrieved [3]. The observed 30-day all-cause mortality of 23.3% is consistent with existing literature describing clinical outcomes in patients with CRE infections [4].

The most common carbapenemase genes in this study were those encoding MBL, especially NDM-1 and NDM-7. Effective antimicrobial therapy options for MBL-producing Enterobacterales are very limited and their management remains particularly challenging [5]. NDM enzymes are established in CRE from the Arabian Peninsula [6, 7]. Given that more than half of the country’s residents originate from the Indian Subcontinent, the high prevalence of NDM in CRE from Qatar is not surprising. NDM genes are commonly co-carried with other carbapenemase genes [5]. In this study, NDM genes were present in all eight isolates that possessed multiple carbapenemase genes.

VIM-producing *Enterobacterales* are endemic in Greece and Eastern Europe, but are otherwise relatively rare [8]. In this study, VIM genes were present in two *E. cloacae* isolates. VIM carbapenemases have also been reported in *K. pneumoniae* and *E. cloacae* from Turkey, Kuwait, and Saudi Arabia [7, 9, 10]. *bla*_IMP-26_ was detected in a single *E. coli* isolate in this study. IMP-producing *Enterobacterales* are endemic in Japan, but are rarely detected in CRE from the Middle East [11].

OXA-48-like β-lactamases are wide-spread in the Arabian Peninsula and the Middle East [6]. Likewise, nearly two-fifth of isolates in this study possessed OXA-encoding genes. Some, but not all, Class D β-lactamases have carbapenemase hydrolyzing activity [12]. Notably, 30.2% of our isolates, all of which possessed OXA-48-like genes, were susceptible to meropenem. As previously well-documented, nonsusceptibility to ertapenem is often the most sensitive indicator of carbapenemase production [13]. Similar to previous reports of frequent co-presence of OXA-48-like enzymes with MBL, seven out of eight isolates with multiple carbapenemase genes in this study possessed genes encoding NDM and OXA-48-like enzymes [12, 14].

OXA-181 and OXA-232 are OXA-48-like carbapenemases. OXA-181 differs from OXA-48 by four amino acids and, like NDM, its origin is epidemiologically linked to the Indian subcontinent [12]. In the Middle East region, OXA-181 was previously identified in CRE from Lebanon and Oman [14, 15]. OXA-232 differs from OXA-48 by five amino acids substitutions, and from OXA-181 by one mutation [12]. It was first reported in *K. pneumoniae* from France, but has since been reported from many parts of the world including South Asia and North Africa [16, 17]. Interestingly, OXA-232 is often present in *K. pneumoniae* ST231 [18]. Four out of 10 OXA-232-producing *K. pneumoniae* in our study belonged to this sequence type. One meropenem-susceptible *E. coli* isolate in this report possessed *bla*_OXA-244_. OXA-244 is a single-point mutation derivative of OXA-48, with weaker carbapenem hydrolyzing activity [12]. First described in *K. pneumoniae* from Spain, OXA-244 is now disseminated in *E. coli* in many countries in Western Europe [19].

An important finding in this study is the presence of KPC-encoding genes in five *K. pneumoniae* isolates, all of which were recovered from patients who had recently received medical care in KPC-endemic regions. KPC enzymes have historically been rare in the Middle East, except in Egypt and Israel [1]. However, reports have recently emerged of their occasional detection in CRE from Saudi Arabia, and the United Arab Emirates [20, 21]. In common with our report, the isolation of KPC-producing *K. pneumoniae* in Saudi Arabia was associated with recent travel to Egypt, a country where KPC-producing *Enterobacterales* are endemic [1, 20]. Travel generally, and medical tourism in particular, has frequently been implicated in international importation of multidrug-resistant pathogens [1]. No carbapenemase-encoding genes were detected in 9.4% of the isolates included in this study. It is likely that resistance in those isolates was mediated by non-carbapenemase mechanisms [22].

Tigecycline, fosfomycin, and aminoglycoside were the most active agents against the study isolates. Despite their in vitro activity, these agents’ clinical utility is limited by reduced efficacy in certain infection sites, excessive toxicity, or rapid emergence of resistance [2]. Ceftazidime-avibactam was recently introduced into clinical practice in Qatar. This β-lactam/β-lactamase inhibitor combination is active against most *Enterobacterales* producing Class A and Class D carbapenemases [2]. However, the introduction of ceftazidime-avibactam to intensive care units in Greece was followed by a change in the pattern of carbapenemases in CRE from *bla*_KPC_ to *bla*_VIM_ predominance [23]. Given the background high prevalence of MBL in CRE in Qatar, such agents should be used judiciously to minimize the risk of selection of MBL producers.

The study isolates belonged to widely diverse sequence types. Only two international multidrug-resistant high-risk clones were relatively common: *E. coli* ST38 and *K. pneumoniae* ST147. However, other important high-risk clones were either infrequent (i.e., *E. coli* ST131, ST405, and ST648; and *K. pneumoniae* ST14, and ST258) or absent (e.g., *E. coli* ST69, ST155, and ST393; and *K. pneumoniae* ST14) [24]. These observations suggest that rather than clonal expansion, sporadic introductions of CRE and plasmid-mediated dissemination of carbapenemases are the main drivers of CRE epidemiology in Qatar. There are numerous examples of inter-species transmission of carbapenemase genes on mobile genetic elements resulting in in vivo emergence of resistance in individual patients, and also driving institutional, national, and international CRE outbreaks [24]. Our findings highlight the epidemiological global interconnectedness of bacterial antimicrobial. Given Qatar’s demographics and its position as a major hub for international travel, the findings described here have regional and global implications. The molecular epidemiology of carbapenem resistance in *Enterobacterales* and its underlying mechanisms should be monitored locally, and interpreted in a global context.

## Conclusion

The most frequent carbapenemases in CRE from Qatar are NDM and OXA-48-like enzymes. KPC, hitherto rare in the Arabian Peninsula, was present in a minority of the isolates, where all cases were associated with recent overseas medical care. CRE from Qatar belonged to diverse sequence types, suggesting limited local clonal expansion. It appears that the epidemiology of CRE in Qatar is largely driven by a combination of sporadic importation and inter-species transfer of mobile genetic elements.

## Supplementary Information

ESM 1 (DOCX 43 kb)

ESM 2 (DOCX 83 kb)
