# Supplementary material for: Molecular characterization of clinical carbapenem-resistant Enterobacterales from Qatar
Source: Eur J Clin Microbiol Infect Dis. 2021 Feb 22;40(8):1779–85. doi: 10.1007/s10096-021-04185-7 (PMC8295067; doi:10.1007/s10096-021-04185-7)
Supplement: Supplementary file 1 — (DOCX 43 kb) [file 10096_2021_4185_MOESM1_ESM.docx]

**Supplementary Data File**

**Molecular characterization of clinical carbapenem-resistant *Enterobacterales* from Qatar**

Fatma Ben Abid,^1,2,3*^ Clement K. M. Tsui,^3,4,5^ Yohei Doi,^6,7^ Anand Deshmukh,^8^ Christi L. McElheny,^6^ William C. Bachman,^6^ Erin L. Fowler,^6^ Ahmed Albishawi,^1,2^ Kamran Mushtaq,^9^ Emad B. Ibrahim,^8^ Sanjay H. Doiphode,^8^ Manal M. Hamed,^8^ Muna A. Almaslmani,^1,2^ Abdullatif Alkhal, ^1,2^ Adeel A. Butt,^3,9^ Ali S. Omrani^1,2^

1. Division of Infectious Diseases, Department of Medicine, Hamad Medical Corporation, Doha, Qatar
2. Communicable Diseases Center, Hamad Medical Corporation, Doha, Qatar
3. Weill Cornell Medicine-Qatar, Doha, Qatar
4. Department of Pathology, Sidra Medicine, Doha, Qatar
5. Division of Infectious Diseases, Faculty of Medicine, University of British Columbia, Vancouver, Canada
6. Division of Infectious Diseases, University of Pittsburgh School of Medicine, Pittsburgh, Pennsylvania, USA
7. Departments of Microbiology and Infectious Diseases, Fujita Health University School of Medicine, Toyoake, Japan
8. Division of Microbiology, Department of Pathology and Laboratory Medicine, Hamad Medical Corporation, Doha, Qatar
9. Department of Medicine, Hamad Medical Corporation, Doha, Qatar
10. Clinical Epidemiology Research Unit, Hamad Medical Corporation, Doha, Qatar

**Methods**

***Study setting***

Hamad Medical Corporation (HMC) provides all hospital-based healthcare services to the population of Qatar. Diagnostic microbiology services for all HMC sites are provided by a central microbiology department. HMC encompasses the following hospitals:

1. Hamad General Hospital (large, acute general and tertiary services)
2. Alwakra Hospital (medium size, acute general hospital)
3. Alkhor Hospital (medium size, acute general hospital)
4. The Heart Hospital (tertiary cardiac services)
5. The National Cancer Care and Research Centre (oncology services)
6. Women’s Wellness and Research Centre (obstetrics, gynaecology and women wellness services)
7. Rumailah Hospital (geriatric and long stay services)
8. Qatar Rehabilitation Institute (rehabilitation services)
9. Ambulatory Care Center (day surgery and interventional diagnostic and therapeutic services)

***Study materials***

Carbapenem-resistant *Enterobacterales* (CRE) isolates from clinical specimens received at HMC Microbiology Department during the period between 1 April 2014 and 30 November 2017 were included. Isolates from samples dated 1 April 2015 thru 31 March 2016 (46 isolates) were included retrospectively, whereas those isolated between 1 April 2016 and 30 November 2017 (103 isolates) were included prospectively. The retrospectively included CRE isolates were retrieved from storage in cryovials at -20°C in HMC Microbiology Department. Clinical data of the patients associated with the isolates were extracted from the electronic health records. National Healthcare Safety Network definitions were used to differentiate infection versus bacterial colonization.^1^

***Identification and susceptibility testing***

Matrix-Assisted Laser Desorption/Ionization-Time of Flight (MALDI-TOF) mass spectrometry (Bruker Corporation, Billerica, Massachusetts) was used for bacterial identification. The initial antimicrobial susceptibility testing for amikacin, cefotaxime, ceftazidime, ciprofloxacin, ertapenem, fosfomycin, gentamicin, meropenem, tigecycline and trimethoprim-sulfamethoxazole was performed on BD Phoenix™ (Becton, Dickinson and Company, Franklin Lakes, New Jersey, United States), using Clinical Laboratory Standards Institute breakpoints.^2^ *Enterobacterales* isolates were included if they were non-susceptible to ertapenem (minimum inhibitory concentration (MIC), >0.5 mg/L) or meropenem (MIC, >1.0 mg/L).^2^ Carbapenem resistance was confirmed using ertapenem disc diffusion method (zone of inhibition diameter of ≤21 mm).^2, 3^ When a CRE species was isolated from multiple sites or on multiple occasions from the same patient, only the first isolate was included.

***Whole genome sequencing (WGS) and analysis***

Genomic DNA were extracted using DNeasy Blood and Tissue Kit (Qiagen, Hilden, Germany). DNA libraries were constructed with a modified Nextera DNA library preparation method (Illumina Inc., San Diego, California, USA), then sequenced on the Illumina NextSeq 550 platform with 2x150 cycles at Microbial Genome Sequencing Center (Pittsburgh, Pennsylvania, USA).^4^ The sequence data was assessed by Fastqc (https://www.bioinformatics.babraham.ac.uk/projects/fastqc/), trimmed by Trim Galore v0.6.0 (http://www.bioinformatics.babraham.ac.uk/projects/trim_galore/), assembled *de novo* using SPAdes v.3.9.0 implemented in shovill (https://github.com/tseemann/shovill), and assessed using QUAST v5.0.2. ^5, 6^ Contigs of smaller size (<500 bp), and with contaminants were excluded after analysis by Kraken v.2.^7^ Sequence type (ST) and antimicrobial resistance genes were predicted from the assembled contigs using multi-locus sequence typing (MLST) (https://github.com/tseemann/mlst), the Plasmidfinder v2.1 and ResFinder v3.2 databases implemented in ABRicate v0.9 (https://github.com/tseemann/abricate), based on >70% coverage and 90% sequence identity.^8^

**References:**

1. CDC/NHSN. National Healthcare Safety Network (NHSN) Patient Safety Component Manual. https://www.cdc.gov/nhsn/PDFs/pscManual/pcsManual_current.pdf.

2. Clinical Laboratory Standards Institute. Performance Standards for Antimicrobial Susceptibility Testing, 30th Edition. CLSI Supplement M100. http://em100.edaptivedocs.net/GetDoc.aspx?doc=CLSI%20M100%20ED30:2020&scope=user.

3. Vading M, Samuelsen O, Haldorsen B et al. Comparison of disk diffusion, Etest and VITEK2 for detection of carbapenemase-producing Klebsiella pneumoniae with the EUCAST and CLSI breakpoint systems. *Clin Microbiol Infect* 2011; **17**: 668-74.

4. Baym M, Kryazhimskiy S, Lieberman TD et al. Inexpensive multiplexed library preparation for megabase-sized genomes. *PLoS One* 2015; **10**: e0128036.

5. Bankevich A, Nurk S, Antipov D et al. SPAdes: a new genome assembly algorithm and its applications to single-cell sequencing. *J Comput Biol* 2012; **19**: 455-77.

6. Gurevich A, Saveliev V, Vyahhi N et al. QUAST: quality assessment tool for genome assemblies. *Bioinformatics* 2013; **29**: 1072-5.

7. Wood DE, Salzberg SL. Kraken: ultrafast metagenomic sequence classification using exact alignments. *Genome Biol* 2014; **15**: R46.

8. Zankari E, Hasman H, Cosentino S et al. Identification of acquired antimicrobial resistance genes. *J Antimicrob Chemother* 2012; **67**: 2640-4.

**Table S1. Study patients by hospital**

| **Hospital** | **Number of Patients** |
| --- | --- |
| Hamad General Hospital | 98 (67.1%) |
| Alwakra Hospital | 18 (12.3%) |
| Alkhor Hospital | 3 (2.1%) |
| The Heart Hospital | 8 (5.5%) |
| The National Cancer Care and Research Centre | 10 (6.8%) |
| Women’s Wellness and Research Centre | 0 |
| Rumailah Hospital | 9 (6.2%) |
| Qatar Rehabilitation Institute | 0 |
| Ambulatory Care Center | 0 |

**Table S2. Antimicrobial susceptibility of the study isolates (number, percent susceptible)**

| **Agent** | **Total susceptible**  **(n = 149)** | ***Escherichia coli***  **(n =38)** | ***Klebsiella pneumoniae***  **(n = 81)** | ***Klebsiella quasipneumoniae^*^***  **(n = 16)** | ***Enterobacter cloacae***  **(n = 7)** | **Others^†^**  **(n = 7)** |
| --- | --- | --- | --- | --- | --- | --- |
| Meropenem | 45 (30.2%) | 19 (50%) | 21(25.9%) | 0 | 2 (28.5%) | 3 (42.9%) |
| Piperacillin-Tazobactam | 17 (11.4%) | 4 (10.5%) | 11 (13.5%) | 0 | 0 | 2 (28.6%) |
| Ciprofloxacin | 34 (22.8%) | 8 (21.1%) | 13 (16%) | 7 (43.8%) | 2 (28.5%) | 4 (57.1%) |
| Amikacin | 108 (72.4%) | 30 (78.9%) | 55 (67.9%) | 15 (93. %8) | 3 (42.9%) | 5 (71.4%) |
| Gentamicin | 75 (50.3%) | 20 (52.6%) | 30 (37%) | 16 (100%) | 3 (42.9%) | 6 (85.7%) |
| Trimethoprim-Sulfamethoxazole | 44 (29.5%) | 8 (21.1%) | 18 (22.2%) | 15 (93.8%) | 1 (14.3%) | 2 (28.6%) |
| Fosfomycin | 126 (84.6%) | 36 (94.7%) | 62 (76.5%) | 16 (100%) | 6 (85.7%) | 6 (85.7%) |
| Tigecycline | 129 (86.5%) | 36 (94.7%) | 65 (80.2%) | 15 (93.8%) | 7 (100%) | 6 (85.7%) |

Figures represent number (%)

**Klebsiella quasipneumoniae* subsp. *quasipneumoniae* (n = 14), and *Klebsiella quasipneumoniae* subsp. *similipneumoniae* (n = 2)

^†^*Klebsiella aerogenes* (n = 2), *Providencia* species (n = 2), *Citrobacter freundii* (n = 1), *Klebsiella oxytoca* (n = 1), *and Proteus mirabilis* (n = 1)

**Table S3. Clinical details for patients with KPC-producing *K. pneumoniae***

|  | **Age and sex** | **Recent medical history** | **Specimen type** | **KPC type** | **Sequence Type** |
| --- | --- | --- | --- | --- | --- |
| Patient 1 | 22 years, male | Kidney transplantation in China | Abdominal Wound | *bla*_KPC-2_ | ST15 |
| Patient 2 | 48 years, male | Liver transplantation in India | Bile | *bla*_KPC-2_ | ST11 |
| Patient 3 | 48 years, male | Crohn’s Diseases with bowel resection and ileostomy in the United Kingdom | Respiratory tract | *bla*_KPC-2_ | ST5029 |
| Patient 4 | 62 years, male | Thymic carcinoma resection in the United States of America | Gastrostomy exit site | *bla*_KPC-3_ | ST258 |
| Patient 5 | 3 months, male | Ketonuria with intensive care admission in India | Respiratory tract | *bla*_KPC-3_ and *bla*_NDM -1_ | Undetermined |
